# Towards Selective Trial Stimulation in Spinal Cord Stimulation: Clinical and Psychological Evidence for an Individualised Implantation Strategy

**DOI:** 10.3390/jcm15145592

**Published:** 2026-07-16

**Authors:** Jakub Wiśniewski, Mateusz Szczupak, Anna Barbara Marcinkowska

**Affiliations:** 1 Department of Neurosurgery, Nicolaus Copernicus Hospital, 80-803 Gdansk, Poland; 2 Department of Anaesthesiology and Intensive Therapy, Nicolaus Copernicus Hospital, 80-803 Gdansk, Poland; szczupak.mateusz@icloud.com; 3 Department of Otolaryngology, Faculty of Medicine, Medical University of Gdansk, 80-210 Gdansk, Poland; 4 Applied Cognitive Neuroscience Lab, Department of Neurophysiology, Neuropsychology and Neuroinformatics, Medical University of Gdansk, 80-210 Gdansk, Poland; 5 2nd Department of Radiology, Medical University of Gdansk, 80-210 Gdansk, Poland

**Keywords:** spinal cord stimulation, neuromodulation, screening trial, patient selection, paresthesia-free stimulation, psychological predictors, closed-loop stimulation

## Abstract

**Background/Objectives**: The mandatory spinal cord stimulation (SCS) trial was established in the era of tonic, paresthesia-dependent stimulation, when pre-implantation outcome prediction was unreliable, validated psychological screening was unavailable, and post-implantation programming options were limited. This narrative review examines whether universal trialing remains justified in the context of contemporary paresthesia-free waveforms, mechanistically informed psychological assessment, and current evidence on the predictive utility of screening trials. The objective was to evaluate the contemporary rationale for selective rather than universal trialing and to propose a structured four-step clinical decision framework integrating contraindication screening, pain phenotype certainty, psychological risk stratification, and centre-level criteria, with the aim of identifying patients in whom a trial is most likely to add prognostic information. **Methods**: PubMed and the Cochrane Library were searched using predefined search strings. Eligible publications included randomised controlled trials (including the TRIAL-STIM RCT), systematic reviews, registry-based cohort studies, health-economic analyses, and consensus guidelines. **Results**: The evidence base comprised randomised controlled trials of trial-versus-no-trial strategies, paresthesia-free waveforms, and closed-loop stimulation (including TRIAL-STIM, SENZA-RCT, EVOKE, and ECHO-MAC), together with large registry and cohort studies, systematic reviews, qualitative patient-experience data, and current consensus guidance. Modern paresthesia-free and physiology-guided paradigms reduce several technical functions historically served by trialing, although the extent varies by waveform. The only randomised trial designed specifically to compare trial-first and no-trial strategies (TRIAL-STIM) found no difference in pain outcomes at 6 or 36 months; however, its single healthcare setting and predominantly paresthesia-based waveform mix limit generalisability. Structured pre-implantation psychological evaluation may provide prognostic information that short-duration trialing cannot replicate. At the same time, contemporary guidelines continue to recommend trialing and several clinically relevant arguments for retaining it persist, including patient experiential learning, identification of screening false negatives, and grey-zone decision-making. **Conclusions**: In carefully selected patients with established neuropathic pain phenotypes and comprehensive pre-implantation evaluation, selective trialing may be clinically reasonable. The proposed four-step framework offers a structured basis for allocating trial stimulation to the patients most likely to benefit from it and should be interpreted as hypothesis-generating pending prospective validation. This review does not advocate abandoning trial stimulation; rather, it argues that its role should be examined within an individualised, phenotype- and risk-stratified pathway.

## 1. Introduction

Spinal cord stimulation (SCS) is an implantable neuromodulation therapy in which electrodes placed in the epidural space deliver controlled electrical fields to the dorsal spinal cord to reduce chronic pain. Since its introduction in 1967 [1], SCS has become an established option for selected refractory neuropathic pain conditions, including persistent spinal pain syndrome type 2 (PSPS-T2, formerly failed back surgery syndrome [FBSS]), complex regional pain syndrome (CRPS), and painful diabetic neuropathy, when conservative and pharmacological management has failed [1,2,3,4,5].

In conventional practice, implantation proceeds in two stages. A temporary externalised system is first placed percutaneously and tested during a screening trial, typically lasting one to three weeks. If the patient reports adequate pain relief, the permanent pulse generator is implanted; if not, the leads are removed [6]. This two-stage approach has accompanied SCS since its earliest clinical use and remains recommended by most contemporary guidelines [6,7].

Stimulation can be delivered using several distinct waveforms. Conventional tonic stimulation produces paresthesia, a tingling sensation that must overlap the painful region, whereas paresthesia-free paradigms, including 10 kHz high-frequency stimulation (HF10), burst stimulation, and fast-acting sub-perception therapy (FAST), achieve analgesia below the perception threshold. Closed-loop systems use evoked compound action potential (ECAP) feedback to adjust stimulation in real time. These modalities differ in placement and programming requirements, which directly affects what a screening trial can and cannot demonstrate.

The mandatory screening trial is a deeply entrenched procedural norm; at first glance, questioning it may appear to question the field itself. This review does not challenge trial stimulation as such. Instead, it asks whether its universal application remains justified after three decades of technological and neurobiological development.

Two strategies must therefore be distinguished at the outset. Selective trialing refers to a strategy in which a screening trial is performed in specific clinical subgroups where it is most likely to add clear prognostic value, rather than as a universal requirement. Direct implantation refers to permanent device implantation without any preceding externalised trial. These are distinct positions: selective trialing preserves the trial for specific patients, whereas direct implantation eliminates it entirely. The latter is a more radical step and requires an evidentiary threshold that has not yet been met. This review therefore examines the rationale for selective trialing rather than universal direct implantation.

The original rationale for universal trialing was sound. Tonic, paresthesia-based SCS required intraoperative confirmation of stimulation coverage; positional adjustments were frequently necessary; and no validated multidomain outcome-prediction framework was available [8,9]. Moreover, in the absence of structured psychological screening, the trial served informally as a behavioural observation window [8]. These practices were rational adaptations to genuinely limited tools. The argument advanced here is contextual, not a retrospective criticism of past clinical practice.

Three developments have since altered that context. Paresthesia-free waveforms have removed the dependence on intraoperative mapping. Mechanistic work linking psychological risk factors to prefrontal–limbic modulation, central sensitisation, and descending pain control has strengthened the rationale for structured pre-implantation psychological assessment [10]. Finally, the only randomised controlled trial designed specifically to compare trial-first and no-trial strategies found no outcome benefit from the trial at six or thirty-six months [11,12].

Each of these developments is examined in detail below, alongside the arguments for continued routine trialing stimulation. The available evidence must be interpreted cautiously because most data remain observational or inferential, and prospective validation of selective trialing strategies is still outstanding. Consequently, the proposed framework should be understood as a research-oriented refinement of candidate selection rather than a direct challenge to current consensus guidance.

Accordingly, this narrative review has three aims: first, to examine whether the original technical and prognostic justifications for universal trialing still hold under contemporary technology and assessment; second, to weigh the arguments for and against selective trialing while fairly representing the case for continued routine use; and third, to propose a structured four-step decision framework for allocating trial stimulation. Because this review is narrative rather than systematic, its methodological limitations are detailed in Section 10. Its intended contribution is to support more individualised candidate selection and to define a testable hypothesis for prospective evaluation, rather than to recommend an immediate change in current practice.

## 2. Materials and Methods

PubMed and the Cochrane Library were searched as the primary databases because of their coverage of randomised trials, systematic reviews, and major neuromodulation journals. Predefined search strings were used, and the full strategy is presented in Table 1. Searches were limited to English-language, peer-reviewed articles published through May 2026. Reference lists of identified studies were hand-searched to identify additional publications not captured by the electronic database search.

The lower date boundary of 2000 was chosen to capture the modern evidence base while spanning the transition from tonic, paresthesia-based stimulation to paresthesia-free and closed-loop paradigms. Foundational earlier literature, including the original 1967 clinical report and the gate-control framework, was retrieved by hand-searching reference lists and is cited where historically necessary. Narrower windows were applied to technology-specific terms (e.g., from 2010 for high-frequency and paresthesia-free stimulation, and from 2015 for evoked compound action potential [ECAP]-controlled closed-loop systems) to reflect the period in which these modalities entered clinical use, as shown in Table 1.

This review is a narrative synthesis and does not meet the criteria for a systematic review or PRISMA-compliant synthesis. Consequently, no formal research question was pre-registered, no inter-rater screening was performed, and evidence was integrated narratively rather than quantitatively aggregated. The search strategy is reported in Table 1 in the interest of methodological transparency, not to imply systematic completeness. The exclusion of Embase is acknowledged as a limitation in Section 10.

Studies were selected narratively by the authors based on relevance to the clinical question, study design, sample size where applicable, and representation of both routine-trialing and selective-trialing perspectives. No formal risk-of-bias assessment was performed, and no study was excluded solely because its findings were unfavourable to the review’s central argument. The synthesis integrates clinical, technological, and psychological evidence.

## 3. Historical Rationale for SCS Trials

**Figure 1:**
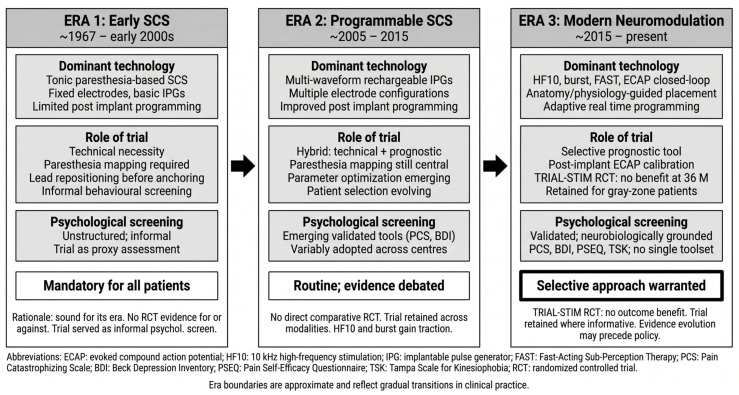
Evolution of spinal cord stimulation practice across three technological eras. Era 1 (1967–early 2000s): tonic, paresthesia-dependent stimulation; the trial served as a technical necessity and informal behavioural proxy. Era 2 (2000s–2015): programmable multi-waveform systems; the trial retained a hybrid technical and prognostic role. Era 3 (2015–present): paresthesia-free (HF10, burst, and FAST) and ECAP-guided closed-loop systems; the original technical rationale for universal trialing substantially reduced. Abbreviations: ECAP, evoked compound action potential; FAST, Fast-Acting Sub-Perception Therapy; HF10, 10 kHz high-frequency spinal cord stimulation.

Mandatory trialing emerged from the constraints of early neuromodulation. The evolution of this practice can be categorised into three technological eras, as summarised in Figure 1.

### 3.1. The First Era (1967–Early 2000s)

Tonic SCS depended entirely on achieving paresthesia overlap with the painful area, a target that was highly sensitive to lead position and impossible to predict before implantation [9,13]. When lead placement was suboptimal, the externalised trial permitted adjustment before permanent anchoring [6]. Without it, a misplaced lead could result in therapy failure that was correctable only by revision surgery. Programming options were limited, making trial response the primary objective data point available before implantation. In addition, psychological screening was not part of standard pre-implantation evaluation, meaning that the trial served informally as a proxy for behavioural suitability [8]. Patient tolerance of stimulation, device management, and sustained adherence were observed during the trial period rather than assessed in advance.

### 3.2. The Second Era (2000s–2015)

Programmable multi-waveform systems expanded the available parameter space. During this period, the trial retained a hybrid role, serving both technical and prognostic functions as programming options increased while pre-implantation prediction remained underdeveloped.

### 3.3. The Third Era (2015–Present)

This era is defined by paresthesia-free waveforms (HF10, burst, and FAST) and evoked compound action potential (ECAP)-guided closed-loop systems, which substantially reduce the original technical rationale for universal trialing. Analysing how each era altered trial functionality is essential to determining whether routine trialing remains warranted in current practice.

## 4. Modern SCS Technology and the Changing Role of the Trial

### 4.1. Paresthesia-Free Waveforms

Three paresthesia-free waveforms now dominate contemporary practice, each differing in mechanism and placement requirements.

HF10 stimulation produces analgesia at 10,000 Hz without conscious paresthesia through mechanisms that include modulation of wide-dynamic-range neurons, GABAergic activation, and glial effects [14]. Lead placement is anatomically guided, making intraoperative paresthesia mapping technically unnecessary. The SENZA-RCT and its 24-month follow-up demonstrated non-inferiority or superiority of HF10 over conventional SCS across back and leg pain outcomes [14,15]. Burst stimulation achieves comparable paresthesia independence through a different mechanism and also uses anatomy-guided placement [16].

FAST (Fast-Acting Sub-Perception Therapy) occupies a distinct mechanistic position and warrants separate consideration. Unlike HF10 or burst stimulation, FAST uses paresthesia-guided field targeting: an optimal stimulation field is first identified using paresthesia coverage, after which amplitude is reduced below the perception threshold for therapeutic delivery [17]. Once the field is established, analgesic onset is rapid, occurring within minutes. Bayerl and colleagues reported durable outcomes in a European real-world retrospective series of 167 patients across 13 centres; at a mean follow-up of 1.6 years, Numeric Rating Scale (NRS) pain scores decreased by 5.1 ± 2.5 points from a baseline of 8.0 ± 1.2 [18]. FAST should therefore not be grouped uncritically with purely anatomy-guided approaches. Its rapid therapeutic latency may reduce the informational yield of extended multi-week externalised trials once an optimal field has been identified, although the initial paresthesia-mapping step retains a role that HF10 and burst stimulation do not require.

### 4.2. ECAP-Guided Closed-Loop Systems and Supraspinal Mechanisms

Closed-loop systems driven by ECAP feedback maintain stimulation automatically within a therapeutic window. This calibration function is not replicated by conventional short externalised trials. The EVOKE double-blind RCT demonstrated superiority over open-loop SCS across multiple outcomes at long-term follow-up [19]; the ECHO-MAC randomised crossover trial found that unwanted stimulation sensation was reduced in 97.6% of participants, with 88.1% expressing a preference for the closed-loop modality [20]. However, these data do not definitively establish that objective post-implantation titration fully replaces the screening function of the trial rather than merely improving it; that is a defensible but substantial inferential step. Taken together, these developments indicate that the technical functions once performed by the trial are increasingly being assumed by the technology itself. This shifts the trial’s residual contribution of the trial from a technical to a primarily prognostic role, without rendering it redundant.

Beyond spinal gate control, modern SCS engages supraspinal mechanisms that bear directly on the trial’s prognostic limitations. Chronic neuropathic pain is associated with maladaptive cortical reorganisation, including expansion of somatosensory representations of the painful region [21], and with central sensitisation: amplification of nociceptive signalling arising from increased membrane excitability, reduced inhibitory tone, and synaptic plasticity within dorsal horn and supraspinal circuits [22]. These processes may evolve over weeks to months. A brief externalised trial cannot capture that trajectory, and direct evidence that SCS reverses central sensitisation or cortical reorganisation in individual patients remains limited [21,22]. This restricts what any short observation window can accurately measure.

### 4.3. Imaging and Surgical Precision

Advances in image-guided implantation have reduced the positional uncertainty that originally motivated the trial’s corrective function. High-resolution preoperative MRI and CT support anatomical targeting of the dorsal columns and identification of epidural anatomy, while intraoperative fluoroscopy, cone-beam CT, and image-fusion techniques improve the accuracy and reproducibility of percutaneous and paddle lead placement [23]. Computational modelling of the electrical field, incorporating patient-specific anatomy and dorsal cerebrospinal fluid thickness, allows stimulation parameters and contact configurations to be estimated before implantation rather than inferred from intraoperative paresthesia mapping [23]. For anatomically guided, paresthesia-free systems, these tools allow leads to be positioned according to defined anatomical landmarks without conscious feedback, narrowing the gap between trial and permanent placement.

These gains are real but bounded. Lead migration remains the most common hardware-related complication [24], anatomical variation and prior spinal surgery can complicate placement, and field-modelling tools are not uniformly available across institutions. The positional argument for trialing is therefore weakened rather than abolished, and it retains greater force where conventional tonic stimulation or technically demanding lead placement is involved (Table 2).

## 5. Clinical Limitations of Routine Trialing

### 5.1. Predictive Validity

A key limitation of routine trialing stimulation concerns a straightforward empirical question: does a short screening trial reliably predict long-term benefit from permanent SCS? The following sections examine whether short-term trial stimulation predicts long-term benefit with sufficient precision to justify universal application.

Earlier systematic reviews documented substantial heterogeneity in long-term outcomes and a consistent tendency for reported pain relief to decline over longer follow-up periods [26,27]. Notably, the TRIAL-STIM randomised comparison found no superiority of a trial-first strategy at six or thirty-six months [11,12]. In the 36-month follow-up, the adjusted between-group difference in pain score was −0.60 (95% CI −1.83 to 0.63), with comparable responder rates (33% in the trial group versus 31% in the no-trial group) and no difference in EQ-5D, indicating that the trial-first strategy conferred no measurable advantage in either pain or health-related quality of life over three years [12]. Corroborating this finding, a Swedish registry study of 411 permanently implanted patients found cumulative explantation rates due to loss of analgesia of 10% at two years and 21% at ten years, suggesting that a substantial proportion of trial successes do not translate into durable benefit, regardless of the trialing strategy used [28].

The mechanisms underlying this discrepancy are related to neuroplasticity. Durable SCS benefit depends on processes such as cortical reorganisation, normalisation of central sensitisation, and engagement of descending control pathways, which require weeks to months to develop [10,22]. A brief externalised trial cannot assess these processes. Instead, it measures an immediate analgesic response that is itself vulnerable to confounding. Trial stimulation introduces novelty, heightened clinical attention, and explicit treatment expectancy: conditions known to activate endogenous opioid and dopaminergic pathways through prefrontal–cingulate and mesolimbic circuits independently of any sustained device effect [10,29]. Trial successes may therefore reflect expectancy-driven responses rather than a patient’s capacity for durable neuromodulation benefit.

Conversely, trial failures pose a parallel problem: they may reflect the technical constraints of the externalised setting, the brevity of the observation window, or the absence of optimal waveform matching rather than genuine non-response to permanent stimulation. North and colleagues demonstrated this directly: using wireless single-stage implants that allowed a full 30-day trial across multiple waveforms, they observed an 88% success rate (substantially higher than rates typically reported with short traditional externalised trials) and an infection rate of only 1% [30]. These findings suggest that some apparent trial failures may reflect features of the trial methodology rather than true non-response to permanent stimulation.

Pre-implantation psychological assessment addresses a different prognostic dimension. Patient mood state is an identifiable predictor of trial outcome: Minnesota Multiphasic Personality Inventory (MMPI) depression and mania subscores differentiated successful from unsuccessful SCS trials with statistical significance in a prospective cohort study; patients reporting adequate pain relief during the trial showed lower depression scores and higher energy levels [31]. Depression remains the most consistently identified psychological predictor of poorer SCS outcomes across cohorts [10,25], and the Swedish registry data confirm that unemployment, a variable closely tracking psychosocial burden, is significantly associated with a lower probability of successful outcome [28]. Structured pre-implantation psychological evaluation, grounded in the neurobiological mechanisms linking prefrontal–limbic function to SCS response [10], provides prognostic information that a short externalised trial cannot replicate or substitute for. The direction of these associations is consistent (higher depression and catastrophising, and lower self-efficacy, track poorer outcomes), but their magnitude and reproducibility vary across cohorts; the predictive evidence and its inconsistencies are examined in detail in Section 7.

### 5.2. Procedural Complications and Patient Burden

The complication burden of SCS is clinically relevant, although trial-specific and implant-related complications should not be conflated. Across SCS studies, overall complication rates of 30–40% have been reported, with infection rates of 3.4–10% and lead migration as the most common hardware-related complication [24]. Trial-phase complication rates may be substantially lower in some series; the wireless single-stage trial reported by North and colleagues observed a 1% infection rate over 30 days, compared with the higher rates associated with traditional externalised percutaneous trials [30].

A further consideration against routine trialing is that a two-stage pathway adds an externalised lead interval, a second anaesthetic event, and additional opportunities for infection and patient burden [11,12,24]. Longer trials carry higher infection rates than shorter protocols [11,12], and a separate anaesthetic event adds procedurally meaningful risk for older or comorbid patients. Qualitative data from the TRIAL-STIM cohort demonstrated a preference for a single-stage procedure (a strong preference in 26 of 31 patients at pre-implantation assessment and 21 of 23 at post-implantation assessment). Patients described the trial phase as disruptive and anxiety-provoking regardless of treatment allocation, although individual cases also showed that the trial experience itself shaped the patient’s final decision to proceed [32].

### 5.3. Risks of Direct Implantation

Omitting the trial step introduces risks of its own. Device explantation carries surgical morbidity and psychological distress. A systematic review of 13,026 patients across 25 studies reported 1882 explantations [33]; reported explantation rates vary depending on the analytic denominator and follow-up structure. Inadequate pain relief was the most common reason for explantation (38%), followed by lead failure (15%) and infection (14%); most explantations occurred within the first year [33]. The Swedish registry data corroborate this pattern: cumulative explantation due to loss of analgesia reached 21% at ten years, with higher age and pre-implantation opioid consumption emerging as significant risk factors [28]. The psychological burden of a failed permanent implant, and the associated erosion of confidence in neuromodulation, are consequences that a correctly negative trial may help avoid. The principal randomised and qualitative studies informing the comparison between trial-first and no-trial strategies are summarised in Table 3.

## 6. Arguments for Continued Routine Trialing

Contemporary consensus guidelines continue to recommend trial stimulation before definitive SCS implantation in most chronic non-cancer pain indications, while acknowledging limitations in the underlying evidence base and emphasising the importance of individual patient assessment [7]. A balanced analysis must therefore address why most experienced clinicians retain this recommendation.

### 6.1. Patient Preparation and Experiential Learning

The screening trial provides unique experiential data that psychometric questionnaires cannot replicate. Over one to three weeks with an active externalised system, patients navigate the device interface, encounter the practical constraints of daily life with an implant, and assess for themselves whether the stimulation sensation is tolerable and consistent with their expectations. During this period, patients form stimulus–response associations, calibrate expectations against lived experience, and identify practical challenges that can be addressed before permanent implantation.

The trial may also identify patients whose unsuitability is not detected through psychological assessment alone. A patient with no flagged risk factors on the Pain Catastrophizing Scale (PCS), Beck Depression Inventory (BDI), or Pain Self-Efficacy Questionnaire (PSEQ) may discover during the trial that the stimulation sensation is aversive despite pain relief; that they cannot reliably manage the programmer because of cognitive or physical factors not captured by questionnaires; or that the constraints of externalised hardware reveal a degree of somatic preoccupation incompatible with long-term therapy engagement [10]. These are genuine false negatives of psychological screening: patients for whom the trial provides information that pre-implantation assessment, however thorough, cannot anticipate. Although the qualitative TRIAL-STIM data predominantly favoured single-stage procedures, they also recorded cases in which the trial experience itself informed the patient’s decision about proceeding [32].

### 6.2. Managing the Grey Zone

A clinically important argument for retaining the trial is that pre-implantation assessment remains imperfect, and a significant proportion of candidates occupy clinically ambiguous territory. A patient with a PCS score of 22, mild-to-moderate depression (BDI 16), and modest self-efficacy concerns (PSEQ 24) cannot be allocated confidently by any existing screening instrument [34,35].

Consider three contrasting profiles. Patient A has a confirmed neuropathic phenotype, PCS below 20, BDI below 10, PSEQ above 40, and no active psychosocial stressors; this profile may reasonably support consideration of direct implantation with a paresthesia-free or closed-loop waveform [34,36]. Patient C has PCS above 30, BDI above 20, active opioid escalation, and an ambiguous pain phenotype; this profile falls outside the operating range of any selective framework and requires either pre-implantation optimisation or a conservative trial-first approach [25,27]. Patient B, the grey-zone candidate, is the case in which the trial retains its strongest individual-level prognostic argument. Real-world behaviour under stimulation, observed over a clinically meaningful period, is a data point that no questionnaire can simulate. Screening instruments have imperfect sensitivity and specificity [25,34,37], and a conservative response to that imperfection retains genuine clinical logic.

**Figure 2:**
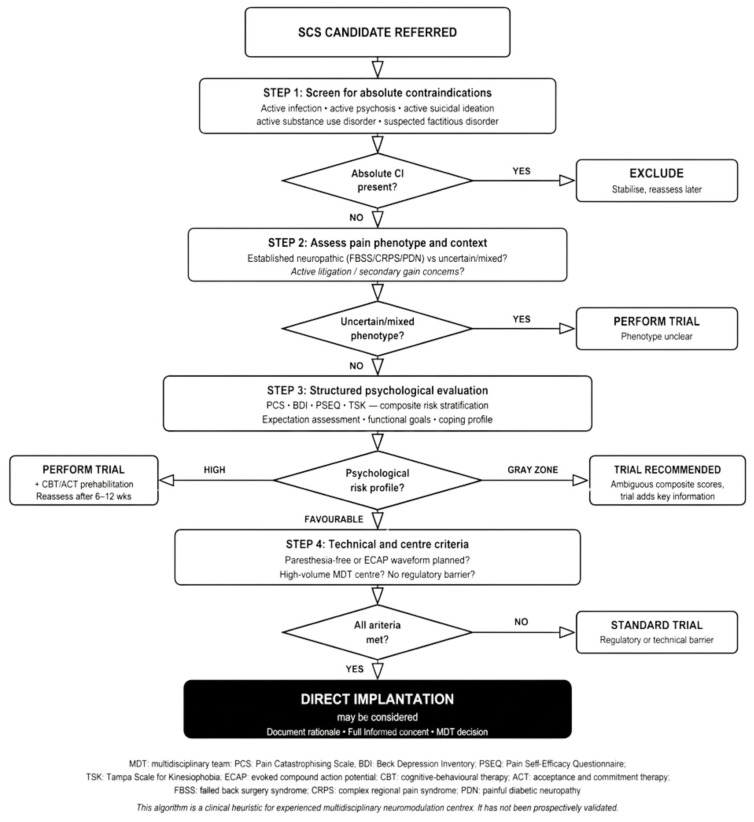
Clinical decision algorithm for selective versus routine trialing in spinal cord stimulation. The algorithm presents four sequential decision points: Step 1, absolute contraindications (hard stop if present); Step 2, pain phenotype certainty (ambiguous phenotype routes to trial); Step 3, composite psychological risk stratification (low risk supports consideration of direct implantation; high risk routes to pre-implantation optimisation or trial-first management; grey zone routes to trial); Step 4, technical and centre-level criteria (paresthesia-free or ECAP-guided waveform availability; multidisciplinary infrastructure). Direct implantation is considered only when all four steps are satisfied. Abbreviations: BDI, Beck Depression Inventory; CRPS, complex regional pain syndrome; ECAP, evoked compound action potential; PCS, Pain Catastrophizing Scale; PDN, painful diabetic neuropathy; PINP, Pre-Implantation Neurophysiological Protocol; PSPS-T2, Persistent Spinal Pain Syndrome Type 2; PSEQ, Pain Self-Efficacy Questionnaire.

The clinical decision algorithm presented in Figure 2 addresses this scenario explicitly: the grey-zone patient is routed to trial stimulation rather than direct implantation because residual prognostic uncertainty is itself the indication for trialing.

### 6.3. Technical Considerations in Paresthesia-Based Systems

The original technical rationale for trial stimulation retains force where conventional tonic stimulation remains the primary modality, or where anatomical, lead-placement, or programming constraints make a short trial clinically informative. In such settings, the trial continues to provide a practical assessment of paresthesia coverage, stimulation tolerability, and early device handling. This is not a marginal consideration: paresthesia-based systems remain in use across many centres, and the evidence base from TRIAL-STIM (conducted predominantly with paresthesia-based waveforms in a UK NHS setting) cannot be extrapolated straightforwardly to paresthesia-free paradigms [11,12].

### 6.4. Synthesis

The following synthesis reflects the authors’ interpretive weighting of the evidence reviewed in Section 3, Section 4, Section 5 and Section 6, rather than a quantitative or guideline-based ranking of arguments. The foregoing arguments remain valid individually but apply with diminishing universality. Patient experiential learning is uniquely informative, but most clearly for grey-zone candidates. Technical uncertainty remains relevant for paresthesia-based systems. A conservative response to ambiguity remains clinically logical wherever pre-implantation assessment is incomplete or equivocal. What these arguments do not support is a categorical requirement to trial every candidate regardless of phenotype, technology, or assessment depth. Recognising their limits motivates a selective rather than blanket application: a structured allocation of trial stimulation to patients in whom it most plausibly adds prognostic value that cannot be obtained otherwise. Standardised psychometric instruments cannot substitute for individualised clinical evaluation, but they remain essential for cohort-level outcome research, where reproducible quantification of psychological status is required to compare groups and study change over time. Their measurement properties, and their limitations for individual-level prediction, are considered in Section 7.

## 7. Psychological Selection in Modern SCS

The neurobiological mechanisms linking psychological status to SCS outcomes have been reviewed in detail in a companion paper by the authors [10]. This section outlines how these psychological dimensions influence descending modulation and central processing, and explains why pre-implantation assessment provides distinct prognostic data that a short externalised trial cannot replicate.

Chronic pain, depression, and anxiety are reciprocally related rather than independent. Persistent pain increases the risk of depressed mood and anxiety, while depression and anxiety amplify pain perception, reduce pain tolerance, and predict poorer treatment response. This bidirectional pattern is attributed in part to shared neural substrates spanning prefrontal, cingulate, insular, and limbic circuits, and to overlapping monoaminergic signalling [22,38,39]. Anxiety and hypervigilance bias attention toward nociceptive input and sustain threat appraisal, whereas depression impairs reward processing and the motivational engagement required for rehabilitation. These interactions are clinically relevant because an invasive analgesic intervention does not act on an isolated nociceptive channel, but on a system in which affective state modulates both pain experience and the capacity to translate analgesia into function. The SCS-specific predictive data should be interpreted against this background.

Pain catastrophising is associated with anterior cingulate cortex and anterior insula hyperactivation, reduced prefrontal top-down inhibitory control, and heightened limbic reactivity [10,22,38,39]. These pathways are directly relevant to descending modulation and may help explain why pre-implantation psychological state is associated with variable SCS outcomes. Depression reflects mesolimbic dysfunction that impairs reward valuation and reinforcement learning, potentially preventing patients from translating device-mediated analgesia into functional recovery [10,39]. Fear-avoidance behaviour may maintain maladaptive cycles even after adequate analgesia is achieved, partly through associative learning mechanisms involving cerebello-cerebral circuits implicated in movement prediction and threat anticipation [40]. In patients with marked hypervigilance, catastrophic appraisal, and fear-avoidance behaviour, device-mediated analgesia may be less likely to translate into durable functional recovery without parallel psychological intervention. Structured questionnaires can identify these risk domains, whereas a short trial can only observe their behavioural consequences.

Multidomain assessment is more defensible than reliance on any single scale. Depression is the most consistently identified psychological predictor of poorer SCS outcomes across systematic reviews and cohort studies [10,25,28]. Pain catastrophising above a threshold of PCS ≥30 was associated with lower pain relief, fivefold higher dissatisfaction rates, and a threefold higher probability of quality-of-life deterioration at six and twelve months after implantation in a prospective multisite study [41]. Catastrophising, anxiety, self-efficacy, coping style, expectation calibration, and secondary gain show clinically relevant but less uniform associations across studies [10,34,36,41]. Therefore, scales such as the PCS, BDI, PSEQ, and Tampa Scale for Kinesiophobia (TSK) should be interpreted as components of a structured risk formulation rather than as a validated deterministic scoring system. High-risk patients still experience a range of outcomes [25,37]. Composite assessment improves clinical confidence but does not eliminate uncertainty.

The instruments most commonly used in SCS outcome research have characteristic strengths and limitations. The PCS is brief, widely validated, and sensitive to a construct with a plausible neurobiological substrate, but its predictive performance is inconsistent across cohorts and its threshold values are not standardised [37,41]. The BDI captures depression, the most consistently replicated predictor, but conflates somatic and affective items in ways that may overlap with pain itself [25]. The PSEQ and TSK address functionally relevant constructs but have been studied less often in SCS-specific samples. The MMPI and MMPI-2-RF provide broad psychopathology coverage and embedded validity scales, but they are long, require trained interpretation, and explain only a modest proportion of outcome variance [31,42]. No single instrument has adequate stand-alone sensitivity and specificity for individual-level allocation; this is the rationale for multidomain assessment and for treating these scales as components of a structured formulation rather than as deterministic scores.

The predictive literature contains several inconsistencies. Poulsen and colleagues found that baseline catastrophising did not predict outcomes in a cohort of 259 patients with long-term follow-up [37]. Bendinger and colleagues found no single variable to be a reliable predictor [34]. MMPI data identify mood state as a significant correlate of trial outcome [31,42], although the variance explained is modest: MMPI depression and mania subscores together accounted for approximately 25% of outcome variance in the Olson cohort [31]. The Swedish registry found that socioeconomic factors, such as education and employment, were among the strongest predictors of patient-reported treatment success, although these variables are not captured by standard psychometric batteries [28]. Psychological assessment therefore remains probabilistic, and comprehensive multidomain evaluation is required before any invasive procedure.

Many relevant risk factors are modifiable. Pre-implantation cognitive-behavioural therapy and acceptance and commitment therapy address catastrophising, fear-avoidance, and expectation calibration through mechanisms that overlap directly with the prefrontal–limbic pathways implicated in SCS response [10]. A patient who is a poor candidate at initial assessment may become a reasonable candidate after targeted psychological preparation. The trial cannot provide this preparation; it can only observe its absence.

### Absolute Contraindications and Risk Stratification

A selective trialing strategy requires unambiguous criteria for when direct implantation should not be considered. The granular stratification of absolute medical and psychological contraindications has been operationalised in detail elsewhere [10]. For the present framework, absolute contraindications to direct implantation include: (i) active psychosis, acute suicidality, or untreated severe major depressive disorder; (ii) active substance use disorder; (iii) somatisation or factitious disorder; (iv) cognitive impairment precluding informed consent or independent device management; and (v) any unresolved condition incompatible with sustained multidisciplinary follow-up. The presence of any absolute contraindication should prompt either deferral of SCS or, where the candidate remains otherwise eligible after intervention, a conservative trial-first pathway.

## 8. A Framework for Selective Trialing

The framework rests on a single organising principle: the value of a screening trial is not fixed but depends on how much prognostic uncertainty remains after pre-implantation assessment. Three premises follow from the preceding sections. First, modern paresthesia-free and closed-loop technology has assumed several technical functions that the trial once performed, so its residual contribution is primarily prognostic rather than technical [14,19]. Second, structured multidomain psychological assessment supplies prognostic information that a short trial cannot replicate, but it does so probabilistically, leaving a group of clinically ambiguous candidates [10,25]. Third, the trial’s incremental value of the trial is therefore greatest where pre-implantation certainty is lowest. The framework operationalises these premises as an ordered sequence in which each decision point either resolves the question or passes an unresolved candidate to the next, with the trial reserved for those in whom uncertainty persists. It is a decision-targeting tool, not an eligibility rule, and it presupposes, rather than replaces, complete multidisciplinary assessment.

The framework presented here is not intended to determine clinical eligibility for SCS, but to identify where trial stimulation may add information beyond multidisciplinary pre-implantation assessment. The evidence reviewed above supports a move from mandatory universal trialing toward an individualised decision grounded in the prognostic function that a trial can serve for a specific patient. The framework is intended for adult candidates being evaluated for SCS implantation for chronic neuropathic pain indications, including PSPS-T2, CRPS, and painful diabetic neuropathy (PDN), in specialist centres with established multidisciplinary protocols; it should not be extrapolated to other indications without separate evidence. Figure 2 presents a stepwise clinical decision algorithm for specialist centres with established multidisciplinary protocols. The algorithm addresses four sequential decision points, each of which must be resolved before proceeding to the next.

Step 1—Absolute contraindications. The contraindications enumerated in Section Absolute Contraindications and Risk Stratification constitute hard stops. Any candidate meeting one or more absolute contraindications is either deferred pending intervention or, where SCS remains appropriate after successful management of the contraindicated condition, routed to a trial-first pathway. Direct implantation should not be considered in this group except in exceptional circumstances and after specialist multidisciplinary review.

Step 2—Pain phenotype certainty. The candidate must have an established neuropathic pain phenotype, confirmed by clinical criteria and, where available, by electrodiagnostic or quantitative sensory testing data within the Pre-Implantation Neurophysiological Protocol [10]. A patient whose pain phenotype remains ambiguous (where the neuropathic contribution is uncertain or where central sensitisation dominates without a clear peripheral generator) belongs in the trial-first pathway regardless of psychological profile. Phenotype uncertainty may itself carry prognostic relevance and is therefore treated here as an indication for trialing.

Step 3—Composite psychological risk stratification. The candidate is assessed using multidomain psychometric evaluation, as detailed in Section 7. Three profiles are possible. A low-risk illustrative profile, such as PCS below 20, BDI below 10, PSEQ above 40, and no active psychosocial stressors, may support consideration of direct implantation when Steps 1 and 2 are satisfied [34,36]. Conversely, a high-risk illustrative profile, for example PCS above 30, BDI above 20, active opioid escalation, low self-efficacy, or active secondary gain, falls outside the operating range of selective trialing and requires pre-implantation optimisation or conservative trial-first management [25,27]. Finally, the grey zone, comprising ambiguous composite scores, routes to the trial pathway. This is not a residual category; in practice, it may represent a substantial proportion of candidates, although its prevalence across centres has not been quantified. The trial’s prognostic value of the trial may be greatest where residual assessment uncertainty is greatest. These numerical profiles are intended as illustrative examples of low-, intermediate-, and high-risk presentations rather than validated decision thresholds. They should not be used as stand-alone criteria for direct implantation or trial allocation.

Step 4—Technical and centre-level criteria. Direct implantation may be considered only where the centre has established multidisciplinary infrastructure, documented experience in SCS implantation, structured outcome monitoring, and access to paresthesia-free or ECAP-guided waveforms that do not depend on intraoperative feedback [7,10]. Where conventional tonic stimulation remains the primary modality, or where post-implantation optimisation capacity is limited, the technical rationale for trialing retains force regardless of the outcomes of Steps 1–3.

The algorithm is intended to allocate trial stimulation to patients in whom it is most likely to add prognostic information, rather than to eliminate trialing. Selective trialing directs the trial’s specific prognostic function (experiential assessment under real-world conditions) to patients for whom pre-implantation evaluation cannot resolve the clinical question; universal trialing applies it indiscriminately regardless of phenotype, technology, or assessment depth. The two approaches are not equivalent in their epistemic claims, and the distinction matters clinically. A sequential patient selection model of this type has been proposed in the neuromodulation literature as a means of operationalising precision pain medicine principles [36], and the present framework builds on that logic while integrating the trial decision as a structured fourth step rather than as the default first step.

The framework has not been prospectively validated. It should not be applied outside high-volume, experienced, multidisciplinary settings, and it should not be used to justify reduced pre-implantation assessment intensity. Selective trialing is not a shortcut. The rigour moves earlier in the pathway—into assessment, phenotyping, and psychological preparation—rather than being delegated to the trial itself.

## 9. Future Directions

The evidence reviewed here supports reconsidering universal trialing as the default strategy and points instead toward a structured research agenda. That agenda has several distinct components, each addressing a different gap in the current evidence base.

The primary research priority is a multicentre randomised controlled trial specifically designed to compare universal and selective trialing strategies in a waveform-stratified population. TRIAL-STIM remains the only published RCT on this question [11,12], and its predominantly paresthesia-based waveform mix and UK NHS setting limit its generalisability to contemporary practice. A well-powered successor trial would require stratification by waveform (paresthesia-free versus paresthesia-based), indication (PSPS-T2, CRPS, and PDN), and psychological risk category; subgroup analyses of grey-zone profiles would be particularly informative. Outcome data at twenty-four months or longer are necessary given the time course of central sensitisation reversal and cortical remapping [21,22]. This requirement is reinforced by the current state of the long-term literature. A recent PRISMA-ScR-informed scoping review mapping SCS outcomes at 24 months or longer found that, although durable benefit is achievable in a substantial subset of patients (most consistently in FBSS/PSPS, and in more recent evidence in painful diabetic neuropathy and closed-loop cohorts), the ≥24-month evidence base remains heterogeneous in outcome definitions, affected by cohort overlap, and inconsistent in its reporting of opioid use and device-maintenance outcomes [43]. A waveform- and indication-stratified successor trial should therefore adopt standardised multidomain long-term outcome reporting rather than rely on the existing fragmented durability literature. Structured collection of complications, explantation rates, patient experience, and functional recovery, rather than pain intensity alone, should be pre-specified as co-primary or secondary endpoints.

Prospective external validation of composite psychometric prediction models is required before any individual-level clinical deployment. The inconsistency in the predictive literature [34,37] reflects, in part, the absence of pre-registered prediction models applied across independent cohorts. High internal accuracy does not establish clinical utility, and the history of prediction modelling in pain medicine offers repeated warnings against premature implementation. Registry-based comparisons between centres with differing trialing strategies, adjusted for case-mix and waveform type, would complement RCT data and are feasible within existing infrastructure: longitudinal datasets such as the Belgian Neuro-Pain^®^ register [44] and the Swedish RAY registry [28] demonstrate how real-world data can refine psychological and clinical predictors of SCS outcomes over time.

Beyond classical psychometric prediction, artificial intelligence (AI) and machine learning (ML) approaches represent a developing research direction for SCS candidate selection and outcome monitoring. Supervised and unsupervised learning approaches applied to prospectively collected cohorts have identified clinically distinct patient subgroups and shown internally validated responder-prediction performance, with an area under the receiver operating characteristic curve (AUC) of 0.71–0.76 [45]. In parallel, objective data from wearable sensors may complement subjective questionnaires for continuous post-implantation monitoring, potentially supporting adaptive models that track functional recovery rather than pain intensity alone [46].

The potential advantages of these approaches include the integration of high-dimensional, multimodal inputs (clinical, psychometric, neurophysiological, device-derived, and behavioural) that exceed the capacity of conventional regression and may help identify patients in whom a trial adds little prognostic information. However, these digital health applications face substantial limitations. Current models are predominantly single-centre or feasibility studies lacking external validation, making them vulnerable to overfitting and outcome-label inconsistency across cohorts. Class imbalance, limited sample sizes relative to feature counts, and the absence of pre-registered, prospectively tested models remain recurrent weaknesses. Model interpretability is also a critical concern; opaque “black-box” models are difficult to incorporate into shared decision-making and informed consent. Consequently, any tool intended to influence implantation decisions requires evaluation as clinical decision-support software within the applicable regulatory framework. AI-assisted selection remains a research objective rather than a current clinical recommendation, and its near-term role is restricted to hypothesis generation and prospective model development.

Structured engagement among academic neuromodulation societies, guideline bodies, and specialist centres is needed to assess whether trialing pathways should be reconsidered as the evidence base evolves. Formal clinical pathway adaptation will likely lag behind evidence evolution by several years, emphasising the need for early, rigorous, and prospectively registered research.

## 10. Limitations

Several limitations constrain the conclusions that can be drawn from this review and should be read as qualifications to the framework proposed in Section 8.

As a narrative review, this work carries the methodological limitations inherent to that design. No protocol was pre-registered, study selection and screening were performed without independent dual review, no formal risk-of-bias or certainty-of-evidence assessment (e.g., GRADE) was undertaken, and data were integrated qualitatively rather than pooled. These features increase susceptibility to selection and confirmation bias and preclude the reproducibility and transparency standards of a PRISMA-compliant systematic review. The synthesis therefore reflects the authors’ interpretation of a purposively assembled body of evidence. Furthermore, the search was conducted only in PubMed and the Cochrane Library; Embase was not searched, and additional databases may have identified further relevant studies. Advocacy bias cannot be entirely excluded, despite explicit efforts to represent both sides of the clinical debate. TRIAL-STIM remains the only published RCT specifically comparing trial-first and no-trial strategies. The six-month primary analysis enrolled 105 randomised participants (TG *n* = 54, NTG *n* = 51) [11], with 66 completing thirty-six-month follow-up (TG *n* = 30, NTG *n* = 36) [12]. Its UK NHS setting, predominantly paresthesia-based waveform mix, and attrition between the primary and follow-up analyses limit generalisability to contemporary paresthesia-free practice in other healthcare systems. Crucially, the trial’s specificity was only 8% (95% CI 1–25%) [11]. Within the definitions used in TRIAL-STIM, a sensitivity of 100% with a specificity of 8% suggests that the trial strategy was far better at identifying likely responders than at excluding likely non-responders. This pattern is consistent with limited capacity to exclude non-responders; whether it generalises to other cohorts and waveforms remains untested.

The SCS population is clinically heterogeneous. Findings from PSPS-T2 and radiculopathy cohorts may not extrapolate to CRPS, PDN, or other indications, and waveform-specific effects on trial utility have not been studied in adequately powered stratified designs. Psychological assessment remains probabilistic, with imperfect and sometimes conflicting predictive consistency across cohorts [34,37]. The composite risk-stratification approach proposed here has not been prospectively validated as a decision tool. Finally, the proposed framework and clinical algorithm require experienced multidisciplinary infrastructure and should not be applied in low-volume or non-specialist settings.

Because of this narrative methodology, the proposed framework must be interpreted as a hypothesis-generating synthesis rather than as a clinical guideline or validated decision rule. The burden-of-evidence threshold for a practice change of this magnitude has not yet been met. The algorithm should not be used to change local clinical pathways without prospective validation, institutional governance, and alignment with applicable consensus recommendations.

## 11. Conclusions

The SCS trial was a well-reasoned clinical practice for its era, and the arguments supporting trial stimulation remain valid in selected contexts. What has changed is the assumption that these arguments apply universally.

Paresthesia-free waveforms have removed dependence on intraoperative feedback. ECAP-guided systems enable objective post-implantation calibration that is not captured by conventional short externalised trials. Neurobiologically grounded psychological screening identifies modifiable prefrontal–limbic and central-sensitisation risk factors before any invasive procedure [10,22]. The only RCT designed specifically to compare strategies found no outcome benefit from trial stimulation at six or thirty-six months [11,12], while its 8% specificity [11] suggests limited ability to exclude non-responders and requires replication in other cohorts.

Conversely, the arguments for continued trialing in specific subgroups remain valid. Patient experiential preparation is uniquely informative for grey-zone candidates. Psychological screening has imperfect sensitivity and specificity, and the trial provides observational data that no questionnaire can replicate. The technical rationale retains force in paresthesia-based systems. These arguments are not discarded; they form the basis on which selective trialing, rather than universal direct implantation, is proposed.

In carefully selected patients with established neuropathic pain phenotypes and comprehensive pre-implantation evaluation, selective trialing may be clinically reasonable. The four-step framework presented in Section 8 and Figure 2 provides a structured basis for that allocation. Selective trialing should not reduce pre-implantation assessment rigour; instead, the rigour shifts earlier in the pathway, specifically into phenotyping, psychological evaluation, and pre-implantation optimisation. It should not be applied outside experienced multidisciplinary centres. It should also be distinguished clearly and consistently from universal direct implantation, a more radical position that requires an evidentiary threshold that has not yet been met.

Future clinical pathways should reflect the evolving evidence base. They should also preserve the individualised multidisciplinary assessment that remains, regardless of trialing strategy, the most defensible foundation for SCS implantation decisions.

Key points:

- The mandatory SCS screening trial originated from technical and prognostic constraints that modern technology has substantially reduced, although not eliminated.
- Paresthesia-free waveforms and ECAP-guided closed-loop systems assume several functions previously served by the trial, particularly intraoperative mapping and post-implantation optimisation.
- The only randomised trial designed to compare trial-first and no-trial strategies (TRIAL-STIM) found no outcome difference at 6 or 36 months, but its setting and waveform mix limit generalisability.
- Structured, multidomain pre-implantation psychological assessment provides prognostic information that a short trial cannot replicate, although it remains probabilistic rather than deterministic.
- Valid arguments for retaining the trial persist for grey-zone candidates, paresthesia-based systems, and centres with limited post-implantation optimisation capacity.
- A four-step framework (contraindications, phenotype certainty, psychological risk, and centre-level criteria) is proposed to allocate trialing selectively; it is hypothesis-generating and requires prospective validation before clinical adoption.

## Figures and Tables

**Table 1:** Literature search strategy.

Study Types Targeted	Limits	Date Range	Search Terms	Database
Original research, RCTs, reviews	All study types; English only	2000–May 2026	spinal cord stimulation trial	PubMed
RCTs, cohort studies, clinically relevant registry studies	English; humans; adults	2000–May 2026	SCS screening trial; direct implantation SCS	
RCTs, prospective cohort, registry	English; clinical studies	2010–May 2026	paresthesia-free stimulation; HF10 spinal cord stimulation	
RCTs, device studies	English; clinical/technical	2015–May 2026	ECAP closed-loop spinal cord stimulation	
Prospective cohort, systematic reviews	English; humans; adults; pain	2000–May 2026	psychological predictors spinal cord stimulation	
Systematic reviews, meta-analyses	Systematic reviews only	All years to 2026	spinal cord stimulation	Cochrane Library
Additional landmark studies not captured by database searches	N/A	N/A	Reference lists of all included studies	Manual search

RCT: randomised controlled trial.

**Table 2:** Historical rationale for mandatory SCS trialing and contemporary clinical counterarguments. ECAP: evoked compound action potential; ECHO-MAC: randomised crossover trial of ECAP-controlled closed-loop SCS; EVOKE: randomised controlled trial of ECAP-controlled closed-loop SCS; FAST: Fast-Acting Sub-Perception Therapy; HF10: 10 kHz high-frequency spinal cord stimulation.

Contemporary Counterarguments	Historical Rationale for SCS Trialing
HF10 and Burst: anatomy-guided placement, paresthesia feedback not required [14,15,16]. FAST uses paresthesia-guided field targeting followed by sub-perception delivery [17,18].	Paresthesia mapping required; trial confirmed stimulation coverage
Improved imaging and computational field modelling reduce positional uncertainty substantially [23].	High positional uncertainty; externalized lead allowed adjustment
Structured evaluation (PCS, BDI, PSEQ, TSK) provides neurobiologically grounded risk stratification [7,10,25].	No validated psychological screening; trial used as informal proxy
ECAP closed-loop systems (EVOKE [19], ECHO-MAC [20]) enable objective, real-time post-implant optimisation.	Limited post-implant programming; failures not correctable
TRIAL-STIM RCT: no outcome difference at 6 or 36 months between trial and no-trial groups [11,12].	Short trial response assumed to predict long-term benefit
Two-stage pathway adds an externalized lead interval and second procedure, increasing potential infection exposure; magnitude depends on trial duration and local practice [11,12,24].	Dual-procedure complications considered an acceptable trade-off

**Table 3:** Key clinical and qualitative studies evaluating trial-first versus no-trial strategies in SCS. CRPS: complex regional pain syndrome; EQ-5D: EuroQol-5D; FBSS: failed back surgery syndrome; MD: mean difference; NRS: numerical rating scale; NTG: no-trial group; PSPS-T2: Persistent Spinal Pain Syndrome Type 2; TG: trial group.

Key Finding	Population/Setting	Design	Study
No NRS difference at 6 months between trial and no-trial groups	*n* = 105 randomised (TG *n* = 54, NTG *n* = 51); neuropathic pain; UK NHS	TRIAL-STIM RCT	Eldabe et al. 2020 [11]
No difference in NRS (adj. MD −0.60, 95% CI −1.83 to 0.63), EQ-5D, or responder rate (33% vs. 31%)	*n* = 66 (30 TG, 36 NTG); PSPS-T2 ~53%, radiculopathy ~19%; UK NHS	36-month follow-up	Eldabe et al. 2023 [12]
Strong single-stage preference (26/31 pre-implant; 21/23 post-implant), regardless of allocation	TRIAL-STIM patients; UK NHS	Qualitative substudy	Chadwick et al. 2021 [32]

## Data Availability

No new data were created or analysed in this study. Data sharing is therefore not applicable.

## Abbreviations

The following abbreviations are used in this manuscript:

Abbreviation	Definition
BDI	Beck Depression Inventory
CRPS	complex regional pain syndrome
ECAP	evoked compound action potential
ECHO-MAC	randomised crossover trial of ECAP-controlled closed-loop SCS
EQ-5D	EuroQol-5D
EVOKE	randomised controlled trial of ECAP-controlled closed-loop SCS
FAST	Fast-Acting Sub-Perception Therapy
FBSS	failed back surgery syndrome
HF10	10 kHz high-frequency spinal cord stimulation
MD	mean difference
MMPI	Minnesota Multiphasic Personality Inventory
NHS	National Health Service
NRS	numerical rating scale
NTG	no-trial group
ODI	Oswestry Disability Index
PCS	Pain Catastrophizing Scale
PDN	painful diabetic neuropathy
PSEQ	Pain Self-Efficacy Questionnaire
PSPS-T2	Persistent Spinal Pain Syndrome Type 2
RAY	Swedish local registry of invasive neurostimulation treatments
RCT	randomised controlled trial
SCS	spinal cord stimulation
SENZA-RCT	randomised controlled trial of HF10 spinal cord stimulation
TG	trial group
TRIAL-STIM	randomised controlled trial comparing trial vs. no-trial SCS strategies
TSK	Tampa Scale for Kinesiophobia
